# Persistent Low Anti-HIV Neutralizing Antibody Titers in HIV/HCV Coinfection Despite HCV Cure: A 5-Year Longitudinal Analysis

**DOI:** 10.3390/vaccines13050539

**Published:** 2025-05-19

**Authors:** Daniel Sepúlveda-Crespo, Víctor Sánchez-Merino, Rafael Amigot-Sánchez, Almudena Rubio-Pérez, Cristina Díez, Víctor Hontañón, Juan Berenguer, Juan González-García, Felipe García, Isidoro Martínez, Eloísa Yuste, Salvador Resino

**Affiliations:** 1 Unidad de Infección Viral e Inmunidad, Centro Nacional de Microbiología, Instituto de Salud Carlos III, Majadahonda, 28220 Madrid, Spain; danisecre@hotmail.com (D.S.-C.); rafael.amigot@isciii.es (R.A.-S.); imago@isciii.es (I.M.); 2 Centro de Investigación Biomédica en Red en Enfermedades Infecciosas (CIBERINFEC), Instituto de Salud Carlos III, 28029 Madrid, Spain; vmsanchez@isciii.es (V.S.-M.); arubio@isciii.es (A.R.-P.); crispu82@gmail.com (C.D.); victor.hontanon@gmail.com (V.H.); jbb4@me.com (J.B.); juangonzalezgar@gmail.com (J.G.-G.); eyuste@isciii.es (E.Y.); 3 Unidad de Inmunidad Humoral y Vacunas Frente al VIH, Centro Nacional de Microbiología, Instituto de Salud Carlos III, Majadahonda, 28220 Madrid, Spain; 4 Unidad de Enfermedades Infecciosas/VIH, Hospital General Universitario Gregorio Marañón, 28007 Madrid, Spain; 5 Instituto de Investigación Sanitaria del Gregorio Marañón, 28007 Madrid, Spain; 6 Unidad de VIH, Servicio de Medicina Interna, Hospital Universitario La Paz, 28046 Madrid, Spain; 7 Instituto de Investigación Hospital Universitario La Paz, 28046 Madrid, Spain; 8 Servicio de Enfermedades Infecciosas, Hospital Clínic de Barcelona, 08036 Barcelona, Spain; fgarcia@clinic.cat; 9 Institut d’Investigacions Biomèdiques August Pi i Sunyer (IDIBAPS), 08036 Barcelona, Spain; 10 Facultad de Medicine, Universitat de Barcelona, 08036 Barcelona, Spain

**Keywords:** HIV, HIV/HCV-coinfection, HCV cure, HIV neutralizing antibodies, humoral immunity

## Abstract

**Background**: Anti-HIV neutralizing antibodies (anti-HIV-nAbs) play a critical role in the immune defense against HIV by preventing viral entry and limiting replication. This study longitudinally evaluated the titers and variability of anti-HIV-nAbs in individuals coinfected with HIV and HCV. Samples were collected at three time points: before starting HCV treatment, one year after completion, and five years post-treatment. **Methods**: A retrospective analysis was conducted on 71 HIV/HCV-coinfected patients who achieved a sustained virologic response following antiviral therapy for HCV. A control group of 41 HIV-monoinfected individuals was also included. Anti-HIV-nAb titers were evaluated by HIV neutralization assays using a panel of six recombinant HIV viruses representing multiple genetic subtypes. Generalized Linear Mixed Models and Generalized Linear Models were used for statistical analysis. *p*-values were adjusted using the Benjamini–Hochberg procedure (*q*-value). **Results**: HIV-neutralizing antibody responses in HIV/HCV-coinfected individuals remained stable over five years following HCV therapy without significant changes (*q*-value > 0.05). The mean neutralization scores remained stable, with baseline scores of 6.1 (95% CI: 5.4–6.7), 6.2 (95% CI: 5.5–6.8) at one year post-HCV therapy, and 6.0 (95% CI: 5.3–6.7) at five years post-HCV therapy. HIV/HCV-coinfected individuals consistently showed lower neutralization scores compared to the control group throughout the follow-up (*q*-value < 0.05). Regression analyses adjusted for age, gender, nadir CD4^+^, and baseline CD4^+^ counts confirmed that the observed differences between HIV-monoinfected and HIV/HCV-coinfected individuals persisted (*q*-value < 0.05) at both the baseline and after HCV therapy completion. **Conclusions**: Successful HCV eradication in HIV/HCV-coinfected individuals did not normalize anti-HIV-nAb titers, which remained consistently lower than those in HIV-monoinfected controls over five years.

## 1. Introduction

Hepatitis C virus (HCV) and human immunodeficiency virus (HIV) coinfection is a significant global health issue due to their shared transmission routes and viral interactions [1]. An estimated 2.3 million people living with HIV (PLWH) worldwide are coinfected with HCV, representing approximately 6.2% of the global PLWH population. This coinfection is most prevalent among people who inject drugs (~80%) [2]. The introduction of direct-acting antivirals (DAAs) has transformed the treatment landscape for HCV, achieving cure rates >95% [3], even in populations with HIV coinfection [4].

Having both HCV and HIV infections is more detrimental to the immune system than having either infection alone. In PLWH, HCV coinfection is a major cause of non-AIDS-related illness and death because it impairs the recovery of CD4^+^ T cells after combination antiretroviral therapy (cART) [1]. Furthermore, in HIV/HCV-coinfected individuals, immune system dysfunction is worsened by chronic inflammation, altered B-cell and T-cell function, and increased immune exhaustion [1].

A DAA-mediated HCV cure in HIV/HCV-coinfected individuals rapidly lowers inflammatory markers and improves plasma cytokines, though not fully normalizing them. Post DAA therapy, the total CD4^+^ and CD8^+^ T-cell counts increase while activated T cells decrease [5]. While HCV-specific CD8^+^ T-cell proliferation may improve, a persistent “exhaustion scar” remains. Regulatory T-cells (Tregs) and myeloid-derived suppressor cells (MDSCs), which are associated with immune suppression, do not fully normalize after HCV cure, suggesting persistent immune dysregulation despite HCV eradication [1].

HIV/HCV-coinfection is characterized by severe B cell dysfunction, an exacerbation of the abnormalities already present in HIV-monoinfection. HIV alone causes altered B cell phenotypes, impaired antibody production, dysfunctional memory, and disrupted cytokine signaling, leading to weakened humoral immunity (e.g., HIV-neutralizing antibodies) and disease progression [6]. HCV coinfection amplifies this dysfunction through several mechanisms: increased B cell apoptosis and turnover [7]; an accumulation of exhausted/dysfunctional memory B cells that impair antibody production and long-term memory [6]; and persistent HCV-driven immune activation, which fuels B cell dysregulation and immune exhaustion [1]. Although HCV treatment and the subsequent sustained virologic response (SVR) in coinfected individuals can alleviate some B cell dysfunction, immune disruptions often persist. This residual dysregulation can compromise the generation of effective immune responses, including HIV-neutralizing antibodies, even after HCV clearance [1]. Thus, despite partial B cell recovery post-HCV treatment, lingering immune abnormalities may hinder effective antibody responses and long-term immunity.

Neutralizing antibodies (nAbs) play a critical role in the immune defense against HIV by preventing viral entry and limiting replication. These antibodies target key functional sites on the HIV-1 envelope glycoprotein (Env). The ongoing interaction between the host immune system and viral escape mechanisms can drive the emergence of broadly neutralizing antibodies (bNAbs). Unlike strain-specific nAbs, bNAbs can neutralize a diverse range of HIV-1 variants; however, they are often ineffective against autologous escape mutants [8].

In HIV/HCV-coinfected individuals, nAb dynamics are more complex. A recent study demonstrated reduced levels of anti-HCV-bNAbs in HIV/HCV-coinfected individuals compared to those with HCV monoinfection [9], illustrating the intricate relationship between coinfection and humoral immunity. However, the effects of a HCV cure on the dynamics of anti-HIV-nAbs remain poorly understood. HCV infection may influence the anti-HIV-nAb response, potentially affecting its efficacy. Furthermore, the long-term persistence and functionality of anti-HIV-nAbs after HCV cure are of particular importance, as they may provide insights into immune reconstitution and the potential need for adjunctive therapies in this population. Addressing this knowledge gap is crucial, especially given the high efficacy of DAAs and the increasing number of individuals achieving HCV clearance.

## 2. Objective

This longitudinal analysis focused on measuring the levels and variability of anti-HIV-nAbs in individuals coinfected with HIV and HCV. Samples were collected at three time points: before initiating HCV treatment and at one and five years following its successful completion.

## 3. Methods

### 3.1. Design Study

This retrospective study examined HIV/HCV-coinfected individuals selected from 15 healthcare centers in Spain (see Appendix A). Individuals were drawn from two prospective observational cohorts: the GeSIDA 3603b cohort [10] and the Escorial cohort [11]. All individuals underwent anti-HCV therapy between 2012 and 2016, receiving either IFN-based regimens (peg-IFN-α/ribavirin or peg-IFN-α/ribavirin/DAAs) or IFN-free DAAs. Following therapy, all individuals achieved SVR, defined as undetectable HCV-RNA levels 12–24 weeks post-treatment, depending on the therapeutic protocol. Participants were followed up for one year and five years after completing HCV treatment; the five-year follow-up occurred between January 2019 and May 2021. At baseline and both follow-up time points, all participants had been receiving stable cART for over six months, consistent with prevailing clinical guidelines at study inclusion. All participants maintained an undetectable HIV viral load (<50 RNA copies/mL) throughout this study. This study excluded individuals with acute hepatitis C, hepatic decompensation, hepatocellular carcinoma, or hepatitis B coinfection. Additionally, participants undergoing immunosuppressive therapy at any of the three time points were not included; as such, treatments could influence antibody responses. HCV RNA levels were not analyzed at the one- and five-year follow-ups; however, clinical confirmation ensured the absence of HCV reinfection.

A total of 71 HIV/HCV-coinfected individuals were included in this study at baseline, who were followed-up after one year (*n* = 71) and five years (*n* = 68) following HCV end-of-treatment (EOT). Thus, only 3 (4.2%) HIV/HCV-coinfected individuals were lost at the last visit, suggesting that there is no systematic bias due to loss to follow-up. As a control group, we utilized data from a previously published cohort of HIV-monoinfected participants with available neutralization data [12]. For appropriate comparison, we established a control group of 41 HCV-seronegative individuals who were receiving cART and had undetectable HIV viral loads. Crucially, this group was matched at baseline to the HIV/HCV-coinfected group in terms of age, sex distribution, current CD4^+^ T cell count (cells/mm^3^), and nadir CD4^+^ T cell count. This same control group was also used at the two subsequent follow-up points.

The study protocol was approved by the Ethics Committee of the Instituto de Salud Carlos III (#CEI PI 23_2011 and #CEI PI 41_2014) and conducted in accordance with the ethical standards of the 1975 Declaration of Helsinki. Prior to enrollment, all participants provided written informed consent.

### 3.2. Clinical Data and Sample Collection

Epidemiological and clinical data were extracted from the databases associated with the two prospective cohorts. This information was collected from medical records using an online form, ensuring compliance with all confidentiality requirements, and subsequently monitored for accuracy.

One and five years following HCV treatment, peripheral blood samples were obtained via venipuncture. These samples were collected in ethylenediaminetetraacetic acid (EDTA) tubes and promptly transported to the HIV HGM BioBank. Within 24 h of collection, the samples underwent processing and were stored at −80 °C until further analysis.

### 3.3. Laboratory Assays

#### 3.3.1. Generation of a Panel of Recombinant Viruses

To generate full-length infectious molecular clones, the *env* region of HIV_NL4–3_ was replaced with env sequences from genetically diverse primary isolates, resulting in a panel of recombinant viruses, as previously described [12,13,14]. Virus strains were chosen based on the V3C3 region, spanning residues 300 to 392 of the gp120 glycoprotein (HxB2 numbering). This panel consists of six replication-competent recombinant viruses representing five distinct genetic subtypes: VI191 (clade A), AC10 (B), 92BR025 (C), 92UG024 (D), and CM244 (E). Additionally, the NL4–3 strain (B) was included as a neutralization-sensitive reference. To ensure specificity in immunoglobulin G (IgG) neutralization assays, an amphotropic vesicular stomatitis virus (VSV) *env* pseudotyped onto an HIV-1 core served as a control virus. Virus strains were selected to represent Tier 2 HIV-1 subtypes commonly circulating in the region, based on prior neutralization profiling studies and availability of well-characterized *Env* clones.

Virus stocks were produced by transfecting HEK293T cells with DNA constructs using the calcium phosphate transfection method, following the manufacturer’s protocol (Promega, Madison, WI, USA). Viral production was assessed by quantifying p24 capsid protein levels in the supernatant via an antigen capture assay (Innogenetics, Ghent, Belgium).

#### 3.3.2. Purification of IgG

IgG was purified from plasma samples using protein A affinity chromatography (Pierce, Rockford, IL, USA). For IgG extraction, 100 μL of plasma was mixed with 500 μL of sterile phosphate-buffered saline, pH 7.4, and purified using a protein A spin column purification kit. To remove smaller molecules, the purified IgG was subjected to extensive dialysis with 50-kDa-cutoff membranes (Spectra/Por; Spectrum Medical Industries, Laguna Hills, CA, USA). As previously recommended, this molecular weight cutoff eliminates residual efavirenz bound to plasma proteins [15]. The flow-through fraction was further diluted to 0.2 µg/mL for neutralization assays.

#### 3.3.3. HIV Neutralization Assays

The purified IgGs were evaluated in triplicate at 0.2 µg/mL concentration using the virus panel described above. Briefly, recombinant viruses (75 µL) were pre-incubated with 25 µL of plasma-purified IgGs at 0.2 µg/mL, corresponding to 1/200 plasma dilution, for 1 h at 37 °C in 96-well plates. Following this incubation, 5 × 10^3^ TZM.bl cells in 100 µL were added to each well. Neutralization assays were conducted in triplicate. After 72 h of infection, luciferase activity in the cell lysates was measured using a 96-well plate luminometer (Orion; Berthold Technologies, Bad Wildbad, Germany). Luciferase activity from cell lysates infected with non-neutralized viruses was 100%. For each sample, a cumulative neutralization score was determined by averaging the neutralization percentages observed across the six viruses included in the panel, as previously described [12]. This cumulative score represents an integrative measure of the breadth and potency of the neutralizing antibody response. A higher score signifies greater breadth and/or stronger neutralizing activity. Employing a cumulative score addresses variability across different viral strains and facilitates standardized comparison among individuals and across timepoints.

Each sample was assayed in triplicate. To assess intra- and inter-assay variability, key samples underwent three independent repetitions in separate runs. HIV-negative plasma was included as a control to monitor specificity and assay performance. Additionally, negative controls and cell-only background wells were incorporated on each plate. Finally, two blinded investigators independently validated all results to ensure consistency and reproducibility.

## 4. Statistical Analysis

Statistical analyses were performed using SPSS v25.0 (IBM Corp., Chicago, IL, USA) and Stata v18.0 (STATA Corp., College Station, TX, USA). GraphPad Prism v10.0 was used to generate plots and graphs.

The statistical analysis involved the use of the Wilcoxon test for comparing paired groups and the Kruskal–Wallis (for multiple independent groups) and Mann–Whitney U (for two independent groups) tests. For the categorical variables, data are summarized as counts and percentages. Continuous variables, in contrast, are presented as medians along with their interquartile ranges (IQR), specifically the 25th to 75th percentiles.

To evaluate changes in anti-HIV-nAbs at three different time points, generalized linear mixed models (GLMMs) were applied, utilizing a gamma distribution and log link function. GLM with a family (gamma) and link (log) was used to evaluate differences in anti-HIV-nAbs between HIV-monoinfected and HIV/HCV-coinfected individuals at the baseline and following successful HCV therapy, adjusted by their age, gender, nadir CD4^+^, and baseline CD4^+^ counts, which were selected by a stepwise selection method (pin < 0.05 and pout < 0.10). GLMM and GLM analyses were performed using bootstrap replication (*n* = 500) and seed (1). Furthermore, the impact of the treatment regimen (IFN-based vs. IFN-free) was evaluated using GLMM and GLM analyses.

These analyses yielded arithmetic mean ratios (AMR), odds ratios (ORs), and 95% CI. To address the problem of multiple comparisons and reduce the risk of false positives, *p*-values were adjusted using the Benjamini–Hochberg procedure for the false discovery rate (FDR), yielding q-values. Statistical significance was defined as a two-tailed *p*-value below 0.05.

### Statistical Power Analysis

Post hoc power analyses were conducted in R v4.4.2 (pwr package) for the main statistical models. For the repeated measures of GLMM assessing the anti-HIV-nAbs response over three time points (baseline, 1 and 5 years) in the HIV/HCV-coinfected group (N = 71), power was calculated to detect a medium-to-large overall effect of time (Cohen’s f = 0.30, ≈d = 0.6 for pairwise), assuming ρ = 0.5 and ε = 0.75 at alpha = 0.05. This analysis yielded 86.6% power, indicating adequate power for the main effect of time. For the GLM comparing the anti-HIV-nAbs response between the HIV/HCV (*n* = 71) and HIV-monoinfected (*n* = 41) groups (alpha = 0.05), a power analysis (alpha = 0.05, two-tailed) indicated approximately 84.7% power to detect a medium-to-large effect size (Cohen’s d = 0.6). Both analyses suggest this study was adequately powered to detect medium-to-large effects.

## 5. Results

### 5.1. Characteristics of the Study Participants

Table 1 presents the baseline epidemiological and clinical characteristics of the 71 HIV/HCV-coinfected individuals treated for hepatitis C. The median age was 51 years, and the majority were male (81.7%). Most individuals reported a history of intravenous drug use (77.5%) and current smoking (66.2%), while only 4.2% reported a current high-alcohol intake (>50 g/day).

Regarding the HIV status, 30.4% had a nadir CD4^+^ count of <200 cells/mm^3^, and 52.1% had a baseline CD4^+^ count of >500 cells/mm^3^. The most common cART regimens consisted of nucleoside reverse transcriptase inhibitors plus integrase inhibitors (NRTI + II; 43.9%), followed by NRTI + non-NRTI (28.8%).

Concerning the HCV status, the median liver stiffness measurement (LSM) was 21.0 kPa, 45.1% of individuals presented an LSM of 25 kPa or higher, and 11.3% were diagnosed with decompensated cirrhosis. Over half of the cohort (57.7%) had received prior HCV therapy. Of those, 63.4% had undergone interferon (IFN)-based therapy, while 36.6% had received IFN-free DAA therapy. HCV genotype 1 (Gt1) was the most prevalent (76.1%), followed by Gt3 (11.3%) and Gt4 (9.9%). The median HCV-RNA viral load was 6.1 log_10_ IU/mL, with 62.9% of individuals exhibiting viral loads of 850,000 IU/mL or higher. Supplementary Table S1 provides further details on HCV genotypes and specific antiviral therapies.

### 5.2. Anti-HIV-nAbs Response

**Figure 1:**
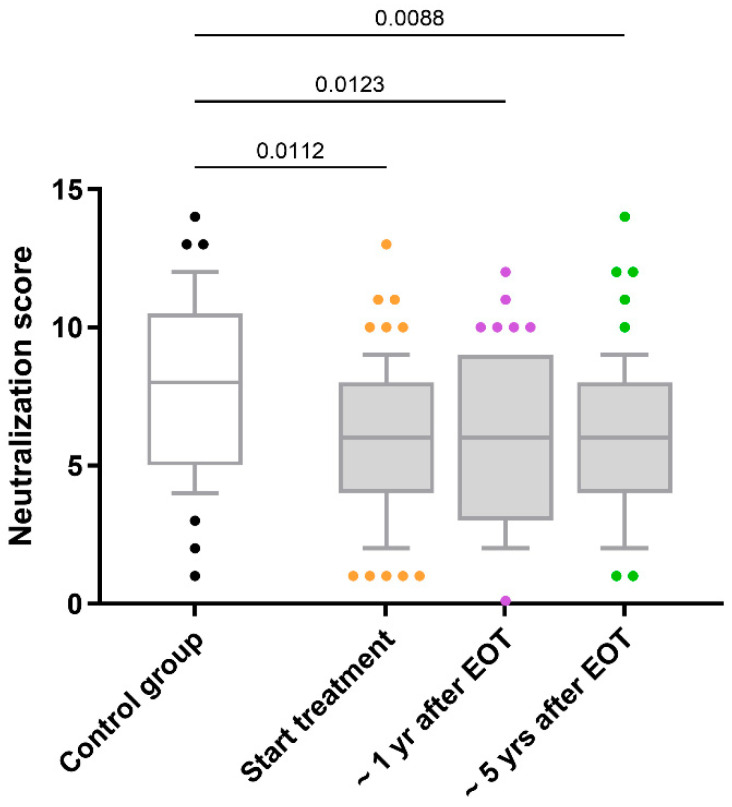
Comparison of the neutralization scores for antibody responses against HIV in HIV/HCV-coinfected individuals. Abbreviations: EOT = end-of-treatment; Yr = year.

HIV-neutralizing antibody responses in HIV/HCV-coinfected individuals remained stable over five years following HCV therapy without significant changes (*q*-value > 0.05; Figure 1). Longitudinal trajectories for each participant (Supplementary Figure S1) further illustrate this overall stability across the cohort, with only a minority exhibiting noticeable fluctuations. Mean neutralization scores remained stable, with baseline values of 6.1 (95% confidence intervals [95% CI]: 5.4–6.7), 6.2 (95% CI: 5.5–6.8) at one-year post-HCV therapy, and 6.0 (95% CI: 5.3–6.7) at five years post-HCV therapy.

Additionally, HIV/HCV-coinfected individuals consistently exhibited lower neutralization scores than the control group throughout the follow-up period (*q*-value < 0.05; Figure 1). Both at the baseline and after the successful completion of HCV therapy, adjusted GLM analyses revealed that the differences between HIV-monoinfected and HIV/HCV-coinfected individuals persisted (AMR < 1; *q*-value < 0.05; Table 2).

### 5.3. Impact of Variables Related to HIV/HCV Coinfection on Anti-HIV-nAbs Response

We evaluated the impact of several variables related to HIV/HCV coinfection on the HIV neutralization score. These variables included the treatment regimen (IFN-based or IFN-free), baseline LSM (≤20 kPa or >20 kPa), baseline HCV RNA viral load (≤850,000 IU/mL or HCV-RNA >850,000 IU/mL), baseline HCV genotype (Non-HCV Gt1 or HCV Gt1), CD4 + T-cell categories (≤200 cells/mm^3^ or >200 cells/mm^3^), and baseline CD4^+^ T-cell categories (≤500 cells/mm^3^ or >500 cells/mm^3^).

**Figure 2:**
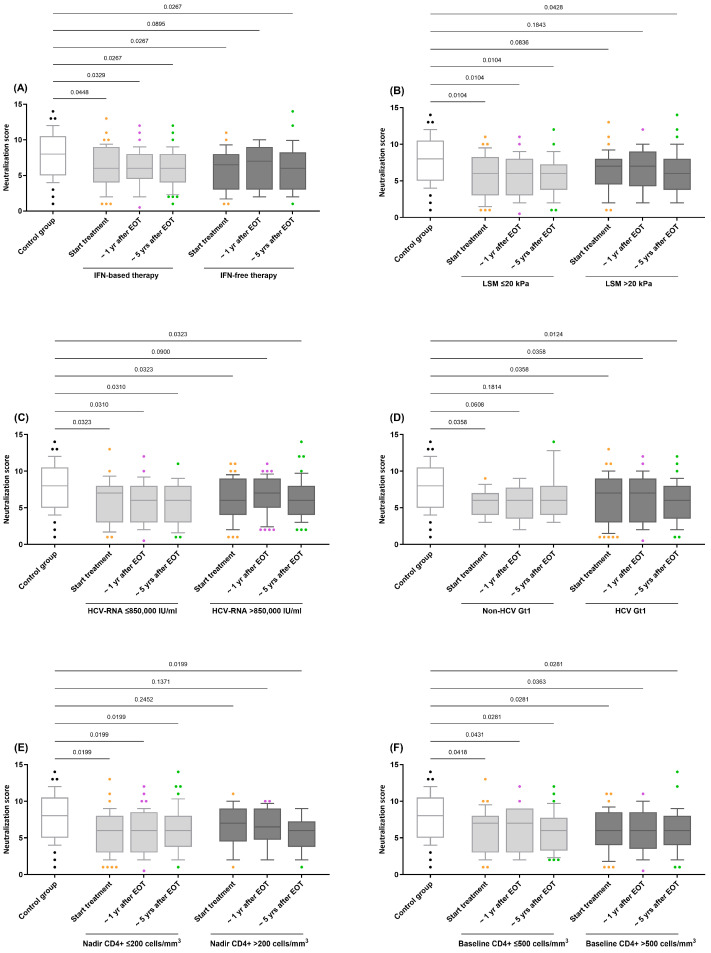
Comparison of the neutralization scores for antibody responses against HIV in HIV/HCV-coinfected individuals stratified by variables related to HIV/HCV coinfection: (**A**) HCV therapy (IFN-based therapy vs. IFN-free therapy); (**B**) LSM (≤20 kPa vs. >20 kPa); (**C**) HCV-RNA (≤850,000 IU/mL vs. >850,000 IU/mL); (**D**) HCV Gt1 (Non-HCV Gt1 vs. HCV Gt1); (**E**) Nadir CD4+ (≤200 cells/mm^3^ vs. >200 cells/mm^3^); (**F**) Baseline CD4+ (≤500 cells/mm^3^ vs. >500 cells/mm^3^). Abbreviations: EOT = end-of-treatment; Yr = year; HIV = Human Immunodeficiency Virus; HCV = Hepatitis C Virus; IFN = Interferon; HCV-RNA = Hepatitis C Virus Ribonucleic Acid.

Within the HIV/HCV-coinfected cohort, none of the evaluated variables significantly impacted the HIV neutralization score in the GLMM analyses (q-value > 0.05). However, when stratified by these variables, practically all resulting subgroups of HIV/HCV participants consistently showed systematically lower neutralization values compared to the control group (q-value < 0.05; Figure 2). This trend was confirmed by adjusted GLM analysis (Supplementary Table S2).

## 6. Discussion

The long-term dynamics of anti-HIV-nAb titers in HIV/HCV-coinfected individuals following successful HCV treatment are illuminated by this valuable longitudinal study. Our primary finding demonstrates that while HCV therapy effectively eliminates HCV, it does not lead to a significant improvement in anti-HIV-nAb titers in HIV/HCV-coinfected individuals. Notably, these anti-HIV-nAb titers remained consistently lower than those observed in HIV-monoinfected individuals throughout the five-year follow-up period. To the best of our knowledge, this is the first study to evaluate the long-term dynamics of anti-HIV-nAbs after a HCV cure in a population of HIV/HCV-coinfected individuals.

Our findings hold significant implications for HIV vaccine development and broader vaccinology regarding the anti-HIV-nAbs response. The persistent sub-optimal anti-HIV nAb titers in HIV/HCV-coinfected individuals, even post-HCV cure, suggest that chronic HCV coinfection induces immune dysregulation, leaving a lasting “immunological scar” on the B-cell compartment. This scar may lead to reduced responsiveness to standard nAb-eliciting HIV vaccine candidates, potentially requiring more potent or tailored strategies. More broadly, our results highlight that the HCV coinfection history and immune reconstitution profoundly modulate the nAb generation capacity, informing the vaccine design and evaluation in populations with complex immunological backgrounds.

The persistence of anti-HIV-nAbs, despite effective cART, is attributed to continuous antigenic stimulation from HIV reservoirs [16,17]. This is supported by studies showing that even individuals with undetectable viral loads maintain detectable anti-HIV-nAbs over extended periods [18,19]. The presence of these reservoirs likely ensures sustained antibody production, which could explain the observed stability over time. This suggests that in coinfected individuals, a HCV cure does not significantly alter this pre-existing immunological equilibrium. Given that HIV-specific neutralizing antibodies play a key role in limiting viral replication and represent a central focus in vaccine research [20,21], understanding the factors influencing their stability in coinfected populations is crucial for future therapeutic strategies.

Chronic HCV infection is known to induce systemic immune activation and dysregulation, which could theoretically affect humoral immunity [22,23]. Some studies have suggested that a HCV cure may restore immune homeostasis, leading to changes in antibody responses [24,25]. However, our findings align with research showing a minimal impact of HCV cure on immune parameters related to HIV [23,26]. Impaired HIV-specific neutralizing antibody responses can persist even after successful HCV eradication in individuals with HIV/HCV coinfection, suggesting that this may cause long-lasting changes in the humoral immune system, which are not fully reversed by clearing HCV. The persistence of these impairments is likely due to durable alterations in B cell function and antibody production.

The observation that anti-HIV-nAb levels remained stable, without a significant increase, for five years post-HCV cure is particularly noteworthy. This stability occurs despite the known association of chronic HIV/HCV coinfection with ongoing B cell dysfunction, including reduced memory B cell frequencies and an expansion of atypical or exhausted B-cell subsets [1]. Several factors may contribute to this persistent impairment of HIV-specific humoral immunity. Firstly, chronic immune activation and exhaustion, driven by both HIV and HCV, can lead to irreversible alterations in B-cell subsets and their functional capacity [27,28]. Secondly, prolonged exposure to HCV antigens might induce epigenetic modifications or alter B-cell receptor repertoires [29,30]. These changes could create a persistent bias towards HCV-specific responses, thereby diminishing HIV-specific immunity and limiting the production of new HIV-specific antibodies even after HCV eradication. Such immunological imprinting may not fully revert after HCV clearance, highlighting the need for targeted interventions to restore effective HIV-specific antibody responses. Alternatively, the absence of an increase in anti-HIV antibody levels could be due to feedback inhibition; pre-existing low levels of anti-HIV antibodies might bind to HIV antigens, thus preventing the stimulation required for further antibody production.

The persistence of diminished anti-HIV-nAb responses in HIV/HCV-coinfected individuals, even after achieving SVR, underscores the complex interplay between these two viral infections and their impact on humoral immunity. This observation is consistent with prior research that has highlighted the significant immunomodulatory effects of chronic HCV infection, which can result in persistent immune dysregulation even after viral clearance [31]. It is plausible, however, that the observed stability of the anti-HIV nAbs could reflect the effectiveness of cART in preserving the overall immune function and preventing the immunological decline in these HIV/HCV-coinfected individuals.

Our study also reinforces the notion that the immune dysregulation observed in HIV/HCV-coinfection is more profound than in HIV monoinfection. The consistent observation of lower anti-HIV-nAb titers in the HIV/HCV-coinfected cohort compared to the HIV-monoinfected control group, even after adjusting for potential confounders, highlights the additive impact of HCV on HIV-related immune dysfunction. This persistent difference underscores the importance of considering the unique immunological challenges faced by HIV/HCV-coinfected individuals.

Our study provides important clinical implications. Although the immediate benefits of HCV cure in HIV/HCV-coinfection are well established, including the reduced risk of liver complications and improved quality of life [32], our study suggests that the humoral immune compartment against HIV remains suboptimal in the long term. The persistent deficit in anti-HIV-nAb responses in HIV/HCV-coinfected individuals may have an impact on HIV disease progression and the potential need for adjunctive immunomodulatory therapies. For instance, strategies aimed at enhancing B-cell function or boosting HIV-specific antibody responses, such as therapeutic vaccination or broadly neutralizing antibody administration, might be particularly beneficial in this population. Furthermore, the long-term stability of nAb titers suggests that these individuals may not experience spontaneous improvements in HIV-specific humoral immunity following HCV cure, emphasizing the need for ongoing monitoring and targeted interventions.

## 7. Strengths and Limitations of This Study

This study has several strengths, including its long follow-up period (five years post-HCV treatment) and the use of a well-defined cohort of HIV/HCV-coinfected individuals. The methodology used to assess the neutralization activity across multiple HIV subtypes provides a robust measure of anti-HIV-nAb function.

However, several limitations must be considered when interpreting our findings. Firstly, the retrospective nature of the study design may introduce potential biases, although we employed strict selection criteria and advanced statistical methods to mitigate these effects. Secondly, the relatively small sample size may limit the study’s power to detect subtle differences in anti-HIV-nAb dynamics. Thirdly, data on the duration of antiretroviral treatment were unavailable for approximately half the patients. Consequently, this variable was excluded from the statistical analysis. While the ART duration might reflect the baseline immune status, we included the nadir CD4^+^ cell count and CD4^+^/mm^3^ values at the baseline as adjustment covariates in the GLM analysis. Moreover, given that all HIV/HCV-coinfected individuals have been on cART for over 6 months, and we have previously shown no correlation between the cART duration and neutralization score after this period [33], the time on cART is expected to have little impact on our study. Fourthly, HCV RNA data were not available during the one- and five-year follow-ups after HCV treatment to confirm the absence of HCV reinfection. However, the absence of HCV reinfection was clinically confirmed during the study period. Fifthly, while we evaluated the breadth and titers of anti-HIV-nAbs, key functional properties like affinity and avidity were not assessed. Therefore, future research involving detailed B-cell phenotyping and functional assays is crucial for a deeper understanding of these antibody responses.

## 8. Conclusions

In conclusion, this study demonstrates that achieving a HCV cure in HIV/HCV-coinfected individuals did not normalize anti-HIV-nAb responses, which remained consistently lower than those in HIV-monoinfected individuals over five years. This suggests that HCV eradication, while beneficial, does not fully restore humoral immunity against HIV. Further research is needed to explore mechanisms underlying this persistent deficit and potential adjunctive therapies to enhance HIV-specific immune responses in this population.

## Figures and Tables

**Table 1:** Overview of the initial epidemiological and clinical characteristics of individuals coinfected with HIV and HCV who received hepatitis C treatment.

Characteristics	Data
**No**	71
**Epidemiological data**	
**Age (years), median [IQR]**	51 [47–54]
**Gender (male), *n* (%)**	58 (81.7)
**Body mass index (BMI, kg/m^2^), median [IQR]**	24.6 [21.5–28.7]
**Smoking status, *n* (%)**	
-Current	47 (66.2)
-Never	5 (7.0)
-Former (>6 months)	19 (26.8)
**Alcohol consumption (>50 g/day), *n* (%)**	
-Current	3 (4.2)
-Never	36 (50.7)
-Former (>6 months)	32 (45.1)
**People who inject drugs, *n* (%)**	
-Current	0 (0)
-Never	16 (22.5)
-Former (>6 months)	55 (77.5)
**HIV-related markers**	
**Prior acquired immunodeficiency syndrome (AIDS) diagnosis, *n* (%)**	3 (4.2)
**Lowest CD4^+^ count/mm^3^, median [IQR]**	135.0 [93.0–234.0]
**CD4^+^ < 200/mm^3^ at nadir, *n* (%)**	21 (30.4)
**Baseline CD4^+^ count/mm^3^, median [IQR]**	518.0 [285.0–731.0]
**Baseline CD4^+^ > 500/mm^3^, *n* (%)**	37 (52.1)
**HIV antiretroviral therapy regimen, *n* (%)**	
**Nucleoside + non-nucleoside reverse transcriptase inhibitors**	19 (26.8)
**Nucleoside reverse transcriptase + integrase inhibitors**	31 (43.7)
**Nucleoside reverse transcriptase + protease inhibitors**	16 (22.5)
**Protease + integrase + non-nucleoside reverse transcriptase inhibitors/maraviroc**	2 (2.8)
**Other**	3 (4.2)
**Liver disease indicators**	
**Liver Stiffness Measurement (LSM, kPa), median [IQR]**	21.0 [12.8–35.0]
-<12.5 kPa	17 (23.9)
-12.5 kPa to 25 kPa	22 (31.0)
-25 kPa to 40 kPa	21 (29.6)
->40 kPa	11 (15.5)
**Hepatic decompensation, *n* (%)**	8 (11.3)
**HCV treatment, *n* (%)**	
**Previous HCV treatment**	41 (57.7)
**Initial HCV treatment**	
-Interferon-based regimen	45 (63.4)
-Interferon-free DAA regimen	26 (36.6)
**HCV-related markers**	
**HCV genotype, *n* (%)**	
-1	54 (76.1)
-3	8 (11.3)
-4	7 (9.9)
-Other/Unknown	2 (2.8)
**Log_10_ HCV-RNA (IU/mL), median [IQR]**	6.1 [5.8–6.6]
**HCV-RNA ≥ 850,000 IU/mL, *n* (%)**	44 (62.9)

Statistics: The values are expressed as the absolute number (percentage) and median (interquartile range). Abbreviations: DAA = direct-acting antiviral; HCV = hepatitis C virus; HCV-RNA = HCV viral load; HIV = human immunodeficiency virus; IQR = interquartile range.

**Table 2:** Differences in neutralization scores between HIV-monoinfected and HIV/HCV-coinfected individuals at baseline and after successful DAA therapy.

	Univariate			Multivariate		
HIV vs. HIV/HCV	AMR (95% CI)	*p*-Value	*q*-Value	aAMR (95% CI)	*p*-Value	*q*-Value
Baseline	0.77 (0.65; 0.91)	0.003	0.004	0.81 (0.67; 0.99)	0.035	0.038
~1 yr after EOT	0.78 (0.66; 0.93)	0.005	0.005	0.82 (0.68; 0.99)	0.038	0.038
~5 yrs after EOT	0.76 (0.64; 0.91)	0.002	0.004	0.79 (0.64; 0.97)	0.026	0.038

Statistics: Data were calculated using GLMs with family (gamma) and link (log). Values are expressed as the AMR, aAMR, and 95% CI. Raw *p*-values and adjusted q-values (*p*-values corrected for multiple testing using the Benjamini and Hochberg false discovery rate procedure) are reported. Statistically significant results are emphasized in bold. Abbreviations: 95% CI = 95% confidence interval; aAMR = adjusted arithmetic mean ratio; DAA = direct-acting antiviral; EOT = end-of-treatment; HCV = hepatitis C virus; HIV = human immunodeficiency virus.

## Data Availability

The datasets generated during and/or analyzed during the current study are available from the corresponding author upon reasonable request.

## Supplementary Materials

The following supporting information can be downloaded at: https://www.mdpi.com/article/10.3390/vaccines13050539/s1, Figure S1: Longitudinal trajectories of neutralization scores for individual participants; Table S1: HCV infecting genotype and antiviral therapies for HIV and HCV in study participants; Table S2: Comparison of Neutralization Scores by HIV Group and HCV Treatment (IFN-based vs. IFN-free) in HIV/HCV-Coinfected Individuals.

## Abbreviations

95% CI	95% confidence interval
AMR	arithmetic mean ratio
bNAbs	broadly neutralizing antibodies
cART	combination antiretroviral therapy
DAA	direct-acting antiviral
EDTA	ethylenediaminetetraacetic acid
*Env*	envelope glycoprotein
FDR	false discovery rate
GLM	generalized linear model
GLMM	generalized linear mixed model
Gt	HCV genotype
HCV	hepatitis C virus
HIV	human immunodeficiency virus
IFN	Interferon
IgG	immunoglobulin G
II	integrase inhibitors
LSM	liver stiffness measurement
MDSC	myeloid-derived suppressor cells
nAbs	neutralizing antibodies
NRTI	nucleoside reverse transcriptase inhibitor
OR	odds ratio
PEG-IFNα	pegylated interferon alpha
PLWH	people living with HIV
RNA	ribonucleic acid
SVR	sustained virologic response
SPSS	statistical package for social sciences
Tregs	regulatory T-cells
VSV	vesicular stomatitis virus

## Appendix A

### Appendix A.1. The GESIDA 3603b Cohort Study Group

*Hospital General Universitario Gregorio Marañón, Madrid:* A Carrero, *p* Miralles, JC López, F Parras, B Padilla, T Aldamiz-Echevarría, F Tejerina, C Díez, L Pérez-Latorre, C Fanciulli, I Gutiérrez, M Ramírez, S Carretero, JM Bellón, J Bermejo, and J Berenguer.

*Hospital Universitario La Paz, Madrid:* V Hontañón, JR Arribas, ML Montes, I Bernardino, JF Pascual, F Zamora, JM Peña, F Arnalich, M Díaz, and J González-García.

Hospital de la Santa Creu i Sant Pau, Barcelona: *p* Domingo and JM Guardiola.

*Hospital Universitari Vall d’Hebron, Barcelona:* E Van den Eynde, M Pérez, E Ribera, and M Crespo.

*Hospital Universitario Ramón y Cajal, Madrid:* JL Casado, F Dronda, A Moreno, MJ Pérez-Elías, MA Sanfrutos, S Moreno, and C Quereda.

Hospital Universitario Príncipe de Asturias, Alcalá de Henares: A Arranz, E Casas, J de Miguel, S Schroeder, and J Sanz.

Hospital Universitario de La Princesa, Madrid: J Sanz and I Santos.

***Hospital Donostia, San Sebastián:*** MJ Bustinduy, JA Iribarren, F Rodríguez-Arrondo, and MA Von-Wichmann.

Hospital Clínico San Carlos, Madrid: J Vergas and MJ Téllez.

**Hospital Universitario San Cecilio, Granada**: D. Vinuesa, L. Muñoz, and J. Hernández-Quero.

Hospital Clínico Universitario, Valencia: A Ferrer and MJ Galindo.

Hospital General Universitario, Valencia: L Ortiz and E Ortega.

***Hospital Universitari La Fe, Valencia:*** M Montero, M Blanes, S Cuellar, J Lacruz, M Salavert, and J López-Aldeguer.

Hospital Universitario de Getafe, Getafe: G Pérez and G Gaspar.

***Fundación SEIMC-GESIDA, Madrid:*** M Yllescas, *p* Crespo, E Aznar, and H Esteban

### Appendix A.2. The ESCORIAL Study Group

***Hospital General Universitario Gregorio Marañón*** (Madrid, Spain): Cristina Díez, Luis Ibáñez, Leire Pérez-Latorre, Diego Rincón, Teresa Aldámiz-Echevarría, Vega Catalina, Pilar Miralles, Teresa Aldámiz-Echevarría, Francisco Tejerina, María C Gómez-Rico, Esther Alonso, José M Bellón, Rafael Bañares, and Juan Berenguer.

***Hospital Universitario La Paz/IdiPAZ*** (Madrid, Spain): José Arribas, José I Bernardino, Ana Delgado, Carmen Busca, Javier García-Samaniego, Víctor Hontañón, Luz Martín-Carbonero, Rafael Micán, María L Montes-Ramírez, Victoria Moreno, Antonio Olveira, Ignacio Pérez-Valero, Eulalia valencia, and Juan González-García.

***Hospital Universitario Puerta de Hierro*** (Madrid, Spain): Elba Llop and José Luis Calleja.

***Hospital Universitario Ramón y Cajal*** (Madrid, Spain): Javier Martínez and Agustín Albillos.

***Fundación SEIMC/GeSIDA*** (Madrid, Spain): Marta de Miguel, María Yllescas, and Herminia Esteban.
