# Supplementary material for: Persistent Low Anti-HIV Neutralizing Antibody Titers in HIV/HCV Coinfection Despite HCV Cure: A 5-Year Longitudinal Analysis
Source: Vaccines (Basel). 2025 May 19;13(5):539. doi: 10.3390/vaccines13050539 (PMC12116166; doi:10.3390/vaccines13050539)
Supplement: Supplementary file 1 [file vaccines-13-00539-s001.zip › vaccines-3579234-supplementary.pdf]

Supplementary Information

Supplementary Table S1. HCV infecting genotype and antiviral therapies for HIV and HCV in study participants

Study participant (#)	HCV genotype	HCV therapy	HIV antiretroviral therapy
**1**	3	SOF + DCV	2 NRTI + II
**2**	1b	SOF + SMV	2 NRTI + II
**3**	1a	SOF + DCV	2 NRTI + II
**4**	4	SOF + LDV	2 NRTI + II
**5**	1a	SOF + DCV	PI + NNRTI + II
**6**	1b	SOF + LDV	PI + II + MVC
**7**	4	SOF + LDV	NRTI + PI
**8**	1b	SOF + LDV	NNRTI + II
**9**	1b	SOF + LDV	2 NRTI + II
**10**	1a	3 DAAs (unspecified)	2 NRTI + II
**11**	4	SOF + DCV	2 NRTI + PI
**12**	1a	SOF + SMV	2 NRTI + II
**13**	1a	SOF + SMV	2 NRTI + II
**14**	1a	SOF + DCV	2 NRTI + PI
**15**	1a	SOF + DCV	2 NRTI + II
**16**	1a	SOF + SMV	2 NRTI + II
**17**	4	SOF + LDV	2 NRTI + II
**18**	4	SOF + LDV	2 NRTI + NNRTI
**19**	1a	SOF + LDV	2 NRTI + II
**20**	1a	SOF + SMV	2 NRTI + II
**21**	1a	SOF + LDV	NNRTI + II
**22**	1a	SOF + LDV	2 NRTI + NNRTI
**23**	4	SOF + LDV	2 NRTI + II
**24**	1a	SOF + DCV + SMV	2 NRTI + PI
**25**	3	SOF + DCV	2 NRTI + PI
**26**	1a	SOF + LDV	2 NRTI + NNRTI
**27**	1b	PEG-IFNα + RBV + PI	2 NRTI + NNRTI
**28**	1a	PEG-IFNα + RBV + PI	2 NRTI + PI
**29**	1a	PEG-IFNα + RBV + PI	2 NRTI + II
**30**	1a	PEG-IFNα + RBV + PI	2 NRTI + PI
**31**	1a	PEG-IFNα + RBV + PI	2 NRTI + PI
**32**	1b	PEG-IFNα + RBV + PI	2 NRTI + PI
**33**	1a	PEG-IFNα + RBV + PI	2 NRTI + II
**34**	1b	PEG-IFNα + RBV + PI	2 NRTI + NNRTI
**35**	3	PEG-IFNα + RBV	2 NRTI + NNRTI
**36**	1a	PEG-IFNα + RBV + PI	2 NRTI + II
**37**	1b	PEG-IFNα + RBV + PI	2 NRTI + II
**38**	MG	PEG-IFNα + RBV + PI	2NRTI + PI
**39**	1a	PEG-IFNα + RBV + PI	2 NRTI + II
**40**	1a	PEG-IFNα + RBV	2 NRTI + NNRTI

**41**	1a	PEG-IFNα + RBV + PI	2 NRTI + II
**42**	3	PEG-IFNα + RBV	2 NRTI + PI
**43**	1a	PEG-IFNα + RBV + PI	2 NRTI + NNRTI
**44**	1b	PEG-IFNα + RBV + PI	II + PI
**45**	IG	PEG-IFNα + RBV + PI	NRTI + PI
**46**	1b	PEG-IFNα + RBV + PI	2 NRTI + PI
**47**	1a	PEG-IFNα + RBV	2 NRTI + II
**48**	IG	PEG-IFNα + RBV + PI	2 NRTI + II
**49**	IG	PEG-IFNα + RBV + PI	2 NRTI + II
**50**	3	PEG-IFNα + RBV	2 NRTI + NNRTI
**51**	3	PEG-IFNα + RBV + SOF	2 NRTI + NNRTI
**52**	IG	PEG-IFNα + RBV + PI	2 NRTI + II
**53**	1a	PEG-IFNα + RBV + PI	2 NRTI + II
**54**	1a	PEG-IFNα + RBV + PI	2 NRTI + NNRTI
**55**	1b	PEG-IFNα + RBV + PI	2 NRTI + NNRTI
**56**	1a	PEG-IFNα + RBV + PI	2 NRTI + NNRTI
**57**	1b	PEG-IFNα + RBV + PI	2 NRTI + PI
**58**	1a	PEG-IFNα + RBV	2 NRTI + II
**59**	1b	PEG-IFNα + RBV + PI	2 NRTI + II
**60**	1b	PEG-IFNα + RBV + PI	2 NRTI + II
**61**	MG	PEG-IFNα + RBV + PI	2 NRTI + NNRTI
**62**	1b	PEG-IFNα + RBV + PI	2 NRTI + NNRTI
**63**	3	PEG-IFNα + RBV	2 NRTI + NNRTI
**64**	4	PEG-IFNα + RBV + PI	2 NRTI + PI
**65**	1a	PEG-IFNα + RBV + PI	2 NRTI + II
**66**	1b	PEG-IFNα + RBV + PI	2 NRTI + NNRTI
**67**	1a	PEG-IFNα + RBV + PI	2 NRTI + PI
**68**	1b	PEG-IFNα + RBV + PI	2 NRTI + NNRTI
**69**	1a	PEG-IFNα + RBV + PI	2 NRTI + NNRTI
**70**	3	PEG-IFNα + RBV	NRTI + NNRTI + II
**71**	1a	PEG-IFNα + RBV + PI	2 NRTI + II

Abbreviations: Study participants **#1-26** are HIV/HCV-coinfected individuals who underwent treatment with IFN-free DAA therapy. Study participants **#27-71** are HIV/HCV-coinfected individuals who received IFNα-based therapy. DAA = direct-acting antiviral; DCV = daclatasvir; HCV = hepatitis C virus; HIV = human immunodeficiency virus; IFNα = interferon alpha; IG = indeterminate HCV genotypes; II = integrase inhibitor; LDV = ledipasvir; MG = mixed HCV genotypes; MVC = maraviroc; NNRTI = non-nucleoside reverse transcriptase inhibitor; NRTI = nucleoside reverse transcriptase inhibitor; Peg-IFNα = pegylated IFNα; PI = protease inhibitor; RBV = ribavirin; SMV = simeprevir; SOF = sofosbuvir.

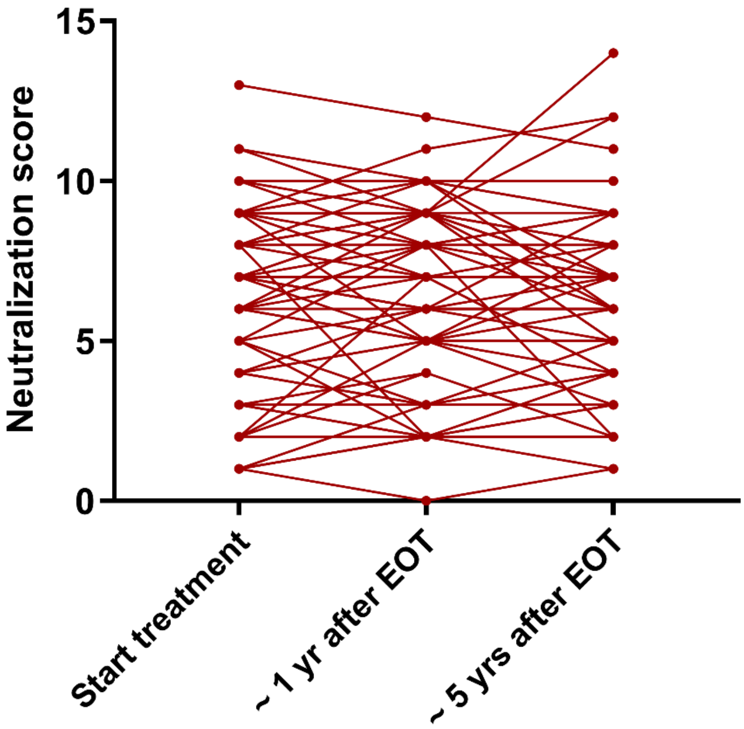

Supplementary Figure S1. Longitudinal trajectories of neutralization scores for individual participants. Neutralization scores measured at multiple time points are shown for each participant. Each line represents a single participant, connecting their neutralization score values over time.

Abbreviations: EOT = end of treatment.

Supplementary Table S2. Comparison of Neutralization Scores by HIV Group and HCV Treatment (IFN-based vs. IFN-free) in HIV/HCV-Coinfected Individuals.

HIV *vs.* HIV/HCV	aAMR (95%CI)	*p*-value	*q*-value
**HCV therapy**			
**Baseline**			
**HIV *vs.* HIV/HCV with IFN-based therapy**	0.22 (0.04; 0.40)	**0.016**	**0.022**
**HIV *vs.* HIV/HCV with IFN-free therapy**	0.33 (0.10; 0.56)	**0.005**	**0.014**
**~ 1 yr after EOT**			
**HIV *vs.* HIV/HCV with IFN-based therapy**	0.24 (0.07; 0.42)	**0.007**	**0.014**
**HIV *vs.* HIV/HCV with IFN-free therapy**	0.25 (0.04; 0.47)	**0.022**	**0.022**
**~ 5 yrs after EOT**			
**HIV *vs.* HIV/HCV with IFN-based therapy**	0.27 (0.10; 0.44)	**0.002**	**0.012**
**HIV *vs.* HIV/HCV with IFN-free therapy**	0.29 (0.04; 0.53)	**0.021**	**0.022**
**Baseline LSM**			
**Baseline**			
**HIV *vs.* HIV/HCV with LSM ≤20kPa**	0.32 (0.12; 0.53)	**0.002**	**0.004**
**HIV *vs.* HIV/HCV with LSM >20kPa**	0.21 (0.02; 0.4)	**0.032**	**0.038**
**~ 1 yr after EOT**			
**HIV *vs.* HIV/HCV with LSM ≤20kPa**	0.33 (0.14; 0.53)	**0.001**	**0.003**
**HIV *vs.* HIV/HCV with LSM >20kPa**	0.17 (-0.02; 0.35)	0.077	0.077
**~ 5 yrs after EOT**			
**HIV *vs.* HIV/HCV with LSM ≤20kPa**	0.32 (0.13; 0.51)	**0.001**	**0.003**
**HIV *vs.* HIV/HCV with LSM >20kPa**	0.23 (0.03; 0.43)	**0.023**	**0.035**
**Baseline HCV RNA viral load**			
**Baseline**			
**HIV *vs.* HIV/HCV with HCV RNA ≤850,000 copies/mL**	0.27 (0.02; 0.52)	**0.032**	**0.033**
**HIV *vs.* HIV/HCV with HCV RNA >850,000 copies/ mL**	0.24 (0.06; 0.43)	**0.010**	**0.018**
**~ 1 yr after EOT**			
**HIV *vs.* HIV/HCV with HCV RNA ≤850,000 copies/ mL**	0.31 (0.08; 0.54)	**0.009**	**0.018**
**HIV *vs.* HIV/HCV with HCV RNA >850,000 copies/ mL**	0.19 (0.02; 0.37)	**0.033**	**0.033**
**~ 5 yrs after EOT**			
**HIV *vs.* HIV/HCV with HCV RNA ≤850,000 copies/ mL**	0.34 (0.09; 0.6)	**0.009**	**0.018**
**HIV *vs.* HIV/HCV with HCV RNA >850,000 copies/ mL**	0.23 (0.05; 0.4)	**0.012**	**0.018**
**Baseline HCV genotype**			
**Baseline**			
**HIV *vs.* HIV/HCV with non-HCV Gt1**	0.31 (0.11; 0.51)	**0.002**	**0.006**
**HIV *vs.* HIV/HCV with HCV Gt1**	0.24 (0.06; 0.43)	**0.008**	**0.012**
**~ 1 yr after EOT**			
**HIV *vs.* HIV/HCV with non-HCV Gt1**	0.28 (0.05; 0.52)	**0.017**	**0.020**
**HIV *vs.* HIV/HCV with HCV Gt1**	0.24 (0.06; 0.41)	**0.007**	**0.012**
**~ 5 yrs after EOT**			
**HIV *vs.* HIV/HCV with non-HCV Gt1**	0.16 (-0.11; 0.42)	0.245	0.245
**HIV *vs.* HIV/HCV with HCV Gt1**	0.31 (0.13; 0.49)	**0.001**	**0.006**
**Nadir CD4$^+$ T-cells**			
**Baseline**			
**HIV *vs.* HIV/HCV with ≤200 cells/mm^3**	0.3 (0.11; 0.49)	**0.002**	**0.009**
**HIV *vs.* HIV/HCV with >200 cells/mm^3**	0.17 (-0.04; 0.39)	0.114	0.114

**~ 1 yr after EOT**			
**HIV *vs.* HIV/HCV with ≤200 cells/mm³**	0.26 (0.08; 0.44)	**0.005**	**0.010**
**HIV *vs.* HIV/HCV with >200 cells/mm³**	0.21 (0; 0.43)	0.052	0.062
**~ 5 yrs after EOT**			
**HIV *vs.* HIV/HCV with ≤200 cells/mm³**	0.24 (0.07; 0.42)	**0.007**	**0.011**
**HIV *vs.* HIV/HCV with >200 cells/mm³**	0.34 (0.12; 0.57)	**0.003**	**0.009**
**Baseline CD4⁺ T-cells**			
**Baseline**			
**HIV *vs.* HIV/HCV with ≤500 cells/mm³**	0.25 (0.05; 0.45)	**0.015**	**0.015**
**HIV *vs.* HIV/HCV with >500 cells/mm³**	0.27 (0.08; 0.46)	**0.006**	**0.014**
**~ 1 yr after EOT**			
**HIV *vs.* HIV/HCV with ≤500 cells/mm³**	0.23 (0.05; 0.42)	**0.015**	**0.015**
**HIV *vs.* HIV/HCV with >500 cells/mm³**	0.26 (0.07; 0.45)	**0.008**	**0.014**
**~ 5 yrs after EOT**			
**HIV *vs.* HIV/HCV with ≤500 cells/mm³**	0.29 (0.1; 0.48)	**0.003**	**0.014**
**HIV *vs.* HIV/HCV with >500 cells/mm³**	0.26 (0.06; 0.46)	**0.009**	**0.014**

Statistics: Data were calculated using GLMs with family (gamma) and link (log). Values are expressed as the AMR, aAMR, and 95% CI. Raw p-values and adjusted q-values (p-values corrected for multiple testing using the Benjamini and Hochberg false discovery rate procedure) are reported. Statistically significant results are emphasized in bold.

Abbreviations: 95%CI = 95% confidence interval; aAMR = adjusted arithmetic mean ratio; HCV = hepatitis C virus; HIV = human immunodeficiency virus; EOT = end of treatment; Yr = year; IFN= Interferon; HCV-RNA = Hepatitis C Virus Ribonucleic Acid.
